# Digital health in musculoskeletal care: where are we heading?

**DOI:** 10.1186/s12891-023-06309-w

**Published:** 2023-03-14

**Authors:** Latika Gupta, Aurélie Najm, Koroush Kabir, Diederik De Cock

**Affiliations:** 1 Department of Rheumatology, Royal Wolverhampton Hospitals NHS Trust, Wolverhampton, UK; 2 City Hospital, Sandwell and West Birmingham Hospitals NHS Trust, Birmingham, UK; 3 Division of Musculoskeletal and Dermatological Sciences, Centre for Musculoskeletal Research, School of Biological Sciences, The University of Manchester, Manchester, UK; 4 Institute of Infection, Immunity and Inflammation, College of Medical Veterinary and Life Sciences, University of Glasgow, Sir Graeme Davies Building Level 4, Glasgow, UK; 5 NHS Royal Alexandra Hospital, Glasgow, UK; 6 Department of Orthopaedics and Trauma Surgery, University Hospital Bonn, Bonn, Germany; 7 Biostatistics and Medical Informatics Research Group, Department of Public Health, Vrije Universiteit Brussel, Brussels, Belgium

**Keywords:** Digital health, Musculoskeletal, Rheumatology, Orthopedics, eHealth, Telemedicine, Virtual consultation, Artificial intelligence

## Abstract

*BMC Musculoskeletal Disorders* launched a Collection on digital health to get a sense of where the wind is blowing, and what impact these technologies are and will have on musculoskeletal medicine. This editorial summarizes findings and focuses on some key topics, which are valuable as digital health establishes itself in patient care. Elements discussed are digital tools for the diagnosis, prognosis and evaluation of rheumatic and musculoskeletal diseases, coupled together with advances in methodologies to analyse health records and imaging. Moreover, the acceptability and validity of these digital advances is discussed. In sum, this editorial and the papers presented in this article collection on *Digital health in musculoskeletal care* will give the interested reader both a glance towards which future we are heading, and which new challenges these advances bring.

Rheumatic and musculoskeletal diseases (RMDs) negatively impact the lives of many, while incurring an outsized economic impact on those working in society. Current treatment strategies improve clinical outcomes but are often labour intensive and patients` demands are high. Moreover, the ratio of patients to health professionals are increasing in many countries, indicating intense pressure on the healthcare system to deliver optimal care [1]. Additionally, options for self-management strategies for patients with RMDs is beyond reach in most understaffed clinics. Digital health may represent a solution for some of these challenges in RMD clinical practice.

Digital health is thus one of the advancing frontiers in musculoskeletal care, which *BMC Musculoskeletal Disorders* set about highlighting when we launched this article collection. The technological spectrum is ever expanding, with regular advances being tested and incorporated into healthcare systems to create efficient care while aiming for the optimal outcome for every patient with. Read within this collection for some exciting research highlights, as the COVID-19 pandemic ushered in an era of remote healthcare [2–4] with telemedicine becoming the new norm [5, 6]. The mass uptake of wearables for routine living has expanded their use for preventive approaches [7, 8]. While the integration of digital solutions into healthcare systems is the inevitable future of musculoskeletal healthcare, data protection laws [9, 10] and formal testing [11, 12] has just begun to come to the fore.

This emerging field refers broadly to health services and information delivered or enhanced through digital technologies [13]. A subpart of digital health, namely mobile health or in short mHealth, refers in general to medical and public health practice supported by mobile devices [14–16]. The past few years have seen exponential increases in mHealth applications, supported by the sudden need of remote care over the COVID-19 pandemic. While the field of mHealth has been limited initially to smartphone apps, the broad range of applications now available for both patients and physicians is on the way to revolutionizing the way we provide routine care. Most of the smartphone apps proposed initially in the literature of musculoskeletal health were focused and limited to the collection of patients reported outcomes [17], subsequently restricting their impact on direct patient care. In addition to their limited functionality, users - especially patients- would rapidly stop as no benefit was perceived on their health; according to a mixed methods study [18]. Therefore, the acceptance of mHealth applications by the various stakeholders in the healthcare system is not always straight forward in clinical practice [19–21]. Use of digital tools to gather self-reported data for patient centered research outlining the patient experience is another emerging area of post pandemic research. An active involvement of patient support groups and patient research partners lends a unique flavor to meaningful patient driven research- *for* the patients and *by* the patients.

Luckily, the revolution in digital health goes further than just applications on smartphones [22, 23]. Perhaps the most advanced field in digital health is the development of analytical methods including machine learning and artificial intelligence (AI) [24–26]. The combination of data from electronic health records, biobanking and/or remote monitoring coupled to the implementation of new methodologies such as AI and gamification will be key for a new era in in daily clinical care for patients living with RMDs [27]. Examples published in this Collection include the recent development of smartphone apps with an image analysis through AI technology to diagnose arthritis or grade structural damage severity on plain radiographs [11, 25]. In addition, apps are now delivering therapeutic interventions such as rehabilitation programs with online personalized coaching linked to connected devices [16, 28, 29]. Virtual -and augmented reality have also started to demonstrate feasibility in telerehabilitation, therapeutic education, self-management advice, and symptom management strategies in the near future for patients with RMDs [30], but we need more standardized methods to evaluate the new technology so that different researcher and physician can exchange data and validate their result [31]. Augmented reality has also started to demonstrate to support surgeons in the operating room [31, 32]. Patient and physician education, and use of open datasets including bibliometrics and altmetrics to track social engagement are other key facets of digital health. The evolving spectrum of cloud-based health approaches offers seamless opportunities to unify global healthcare.

## Reliability and validity

One of the core tenets of robust science is that it is accomplished through validating original findings. Only then can a model become reproducible, generalizable and potentially translated into clinical practice [33]. Prediction tools that often require validation the most, but are not often completed, include prognostic models, trial interventions, nomograms, decision trees, risk scores and web applications.

Original research is characterised by specific conditions (e.g. hospital, patient population), and a tool’s performance is generally suboptimal in a new cohort compared to the population it was developed on [34]. Thus, validation assumes an important role in establishing accuracy of new tools, i.e. testing in another context to see if the original prediction and findings reproduce to a satisfactory level. Validation comes in many forms - from internal that makes use of the original data, **to temporal** which probes new patients in the same cohort at a later timepoint. The most rigorous and therefore most trustworthy is **i**ndependent validation, say an external cohort in a different country or disease group. Only then can one verify the widespread usefulness of said approach and recommend its implementation into clinical practice [35].

In this *Digital health in musculoskeletal care* article collection, as expected in an emerging field, some technologies are still in the initial piloting phase. These include the “BackRx, a personalized mobile phone application for discogenic chronic low back pain” [16] and remote sample of biomarkers of inflammation in patients with RMDs [36]. Independent validation is typically not required at this early stage and more about determining feasibility and safety. Some others aimed to achieve internal validation by determining the intra-class correlation coefficients, intra- and/or inter-observer reliability of their digital applications [12, 14, 15, 22, 37]. Others facilitated generalizability by incorporating a multi-centre prospective study design to assess their artificial intelligence and smartphone app interventions, respectively [11, 26]. Another even utilized temporal validation, when a new online program during orthopedic trauma surgery outpatient clinics was verified in a late cohort after the consultation was established [3].

Other articles in this collection did not include validation methods, and future digital health studies are strongly encouraged to validate the original findings. Compliance improves the impact of one’s research, and guidelines like REMARK (Reporting Recommendations for Tumor Marker Prognostic Studies) have introduced mandates for independent validation in the other research field [38].

To conclude, many digital health technologies have advanced over past years with the potential to impact positively patient care in a near future. Mobile health applications, and integrative analyses by machine learning or artificial intelligence seem to be the precursors that will enable rheumatic and musculoskeletal health care providers to deliver health interventions in a patient-tailored way. These techniques and practices need to be complemented by informative and evaluation processes to prevent an increase in health professionals work burden; and similarly, to avoid overwhelming staff with alerts from digital applications instigated by artificial intelligence.

We live in a time of Big Data coming from various sources such as electronic healthcare records, mHealth applications, biobank and -omics data. Likewise, methodologies such as artificial intelligence enable analysis and provide hope for continuous optimization of clinical care. However, many challenges still lay ahead before many of the digital health solutions currently used in research will make their way into practice.

While diligent patient care assumes the forefront, a wider application of creative solutions for research and education are important collaterals in the digital evolution. This collection offers a glimpse into the break of dawn in digital RMD evolution.

## Data Availability

Not relevant.

## Abbreviations

AI: Artificial intelligence
RMDs: Rheumatic and musculoskeletal diseases
